# Gaussian Multiscale Aggregation Applied to Segmentation in Hand Biometrics

**DOI:** 10.3390/s111211141

**Published:** 2011-11-28

**Authors:** Alberto de Santos Sierra, Carmen Sánchez Ávila, Javier Guerra Casanova, Gonzalo Bailador del Pozo

**Affiliations:** Group of Biometrics, Biosignals and Security, Universidad Politécnica de Madrid, Campus de Montegancedo s/n, 28223 Pozuelo de Alarcón, Madrid, Spain; E-Mails: csa@cedint.upm.es (C.S.A.); jguerra@cedint.upm.es (J.G.C.); gbailador@cedint.upm.es (G.B.P.)

**Keywords:** hand biometrics, multiscale aggregation, image segmentation, image processing, biometrics, security

## Abstract

This paper presents an image segmentation algorithm based on Gaussian multiscale aggregation oriented to hand biometric applications. The method is able to isolate the hand from a wide variety of background textures such as carpets, fabric, glass, grass, soil or stones. The evaluation was carried out by using a publicly available synthetic database with 408,000 hand images in different backgrounds, comparing the performance in terms of accuracy and computational cost to two competitive segmentation methods existing in literature, namely Lossy Data Compression (LDC) and Normalized Cuts (NCuts). The results highlight that the proposed method outperforms current competitive segmentation methods with regard to computational cost, time performance, accuracy and memory usage.

## 1. Introduction

Hand biometrics is receiving an increasing attention at present because of their huge applicability in daily scenarios and the relation between user acceptance and identification/verification rates [1,2].

The characteristics of this biometric technique in terms of non-invasiveness and acceptability highlight the fact that hand biometrics could be a proper and adequate biometric method for verification and identification in devices like PC or mobile phones, since hand biometrics system requirements are easily met with a standard camera and hardware processor.

However, as applications requiring hand biometrics tends to contact-less, platform-free scenarios (e.g., smartphones [3]), hand acquisition (capturing and segmentation) is being increased in difficulty. In other words, hand biometrics is evolving from constrained and contact-based scenarios [4,5] to opposite approaches where less collaboration is required from individuals [3,6], providing non-invasive characteristics to this biometric technique, and thus, improving its acceptability.

Consequently, image pre-processing becomes compulsory to tackle with this problem, by providing an accurate segmentation algorithm to isolate hand from background, whatever its nature, and independent from environment and illumination conditions.

Thus, a segmentation method is proposed able to isolate hand from different background, regardless the environmental and illumination conditions.

The proposed approach is based on multiscale aggregation, gathering pixels along scales according to a given similarity Gaussian function. This method produces an iterative clustering aggregation, providing a solution for hand image segmentation with a quasi-linear computational cost and an adequate accuracy for biometric applications.

The method has been tested with a synthetic image database, with around 408,000 images considering different backgrounds (e.g., soil, skins/fur, carpets, walls or grass) and illumination environments, and compared to two competitive approaches in literature in terms of image segmentation. These approaches are named Lossy Data Compression (LDC) [7] and Normalized Cuts (NCut) [8].

Finally, the layout of the paper remains as follows: Section 2 provides and overview on the current literature, describing the proposed method under Section 3. The database involved in evaluation is presented in Section 4, together with the results, presented in Section 5, providing conclusions and future work in Section 6.

## 2. Literature Review

Segmentation is an important research field in image processing [9], essential in biometric techniques involving image-based data acquisition like hand geometry [10], palmprint [11], hand vein [12], face [13], iris [14], ear [15], gait [16] or handwriting [17].

In fact, the overall performance in terms of identification accuracy relies strongly on the result provided by the segmentation and pre-processing procedure.

Concerning hand-based biometrics, segmentation has received little attention in early works, provided that initial approaches carry out the acquisition procedure in a constrained and homogeneous background [4,18]. This background was selected so that hand segmentation is a trivial task by simple thresholding.

However, as hand biometrics is evolving from contact and peg-based approaches to completely contact-less, peg-free and platform independent scenarios, hand segmentation is increasing its difficulty and complication [6,19,20].

Several approaches in literature tackle with this problem by providing non-contact, platform-free scenarios but with constrained background, usually employing a monochromatic color, easily distinctive from hand texture by means of simple image thresholding [21–23]. More realistic environments propose a color-based segmentation, detecting hand-like pixels either based on probabilistic [24], clustering methods [25] or edge detection [4,5,20].

A possible solution for unconstrained and non-homogeneous backgrounds is a segmentation method based on multiscale aggregation [26–30], inspired on the well-known Normalized Cuts approach [8].

The most common applications of this approach consider image segmentation and boundary detection based on texture [29,31], providing accurate results when compared to human segmentation and other competitive approaches in literature [32].

The results obtained by multiscale aggregation in the fields of unsupervised image segmentation are certainly promising [32], and the application of this method for hand segmentation has been recently proposed [3].

Nonetheless, several aspects must be improved in terms of computational cost and memory usage efficiency [3,30,32]. In fact, these methods are strongly dependent on the number of pixels in an image, and only small images are supported. This limitation was partially solved [3,30], providing a quasi-linear segmentation method, described in detail in the following section.

## 3. Gaussian Multiscale Aggregation

The proposed approach attempts to provide an accurate segmentation of a colour hand image. The algorithm strategy consists of aggregating similar nodes according to a specific criteria along different scales until a given goal is met, ensuring that aggregated nodes within segments verify certain properties.

First step of the algorithm consists of providing a particular structure to the amount of elements within the image. Likewise to other methods [30], the proposed algorithms assumes that a given image *I* can be represented by a graph 𝒢 = (𝒱, ℰ) where nodes in 𝒱 represent pixels in the image and edges in ℰ stands for the structure provided to the set of nodes.

In this approach, the structure on the first scale is assumed to be a 4-neighbourhood strategy, while for subsequent scales, structure is provided by means of Delaunay triangulation [33].

In addition, each node is represented by a similarity function denoted by $\varphi_{v_{i}}^{\left[s\right]}$, where *v_i_* ∈ 𝒱 designates a node in graph 𝒢 and *s* indicates the scale the element *v_i_* belongs to. This similarity function is described in terms of relative measures with respect to intensity average and standard deviation.

More in detail, $\varphi_{v_{i}}^{\left[s\right]}$ is represented by a gaussian distribution 𝒩(*μ*, *σ*) where *μ* and *σ* specify the average and standard deviation neighbour intensity, provided the 4-neighbourhood structure.

Thus, similarity functions leads to the concept of likelihood between nodes in connecting edges, providing a definition of weights within graph 𝒢.

Given a graph 𝒢 = (𝒱, ℰ), the similarity among pair of nodes is provided by means of weights 𝒲, which are defined for each scale *s* as:

(1) $${𝒲}_{i,j}^{\left[s\right]}=\int_{\alpha }\sqrt{\varphi_{v_{i}}^{\left[s\right]}\varphi_{v_{j}}^{\left[s\right]}}d\alpha$$

where *v_i_*, *v_j_* ∈ 𝒱, ∀*i*, *j* and $\varphi_{v_{i}}^{\left[s\right]}$, $\varphi_{v_{j}}^{\left[s\right]}$ represent the similarity function for nodes *v_i_* and *v_j_*, respectively. In addition, *α* stands for the selected colour space, which in this paper corresponds to the *a* layer of the CIELAB (CIE 1976 L*,a*,b*) colour space, due to its ability to describe all visible colors by the human eye [9].

**Figure 1.**
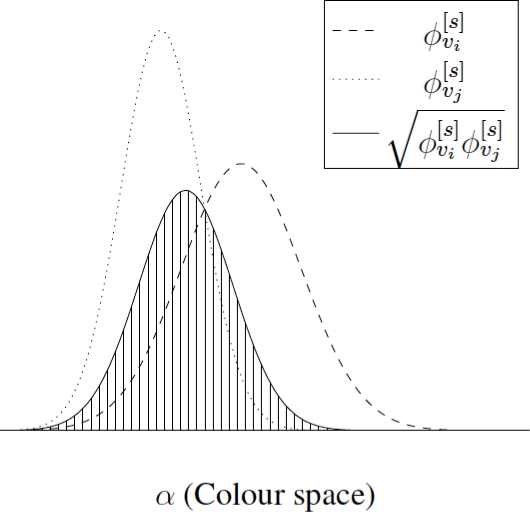
Visual representation of two functions *φ*^[^*^s^*^]^ and the weighted ${𝒲}_{i,j}^{\left[s\right]}$ associated (striped region).

Figure 1 represents two functions *φ*^[^*^s^*^]^ associated to a pair of nodes *v_i_* and *v_j_*, showing the weight associated to their similarity (striped region). The higher the similarity between both nodes, the bigger the striped region.

Therefore, graph 𝒢 = (𝒱, ℰ, 𝒲) contains not only structural information on a given scale *s* but also relational details about the similarity of each node neighbourhood.

Furthermore, 𝒲_*i*,*j*_ can be regarded as the weight associated to edge *e*_*i*,*j*_, so that 𝒲_*i*,*j*_ = 𝒲(*e*_*i*,*j*_). Notice that weights are not defined for each pair of nodes in 𝒱, but only for those pairs of nodes with correspondence in edge set ℰ.

Some properties can be extracted from the definition of 𝒲_*i*,*j*_ ∈ 𝒲 as the similarity between two nodes *v_i_* and *v_j_*, then 𝒲_*i*,*j*_ satisfies ∀*i*, *j*:

1. 𝒲_*i*,*j*_ ≥ 0
2. 𝒲_*i*,*j*_ = 𝒲_*j*,*i*_
3. 𝒲_*i*,*j*_ = 1 ↔ *φ_i_* = *φ_j_*

Property (1) results from the definition given by Equation (1), since the integration of two non-negative functions provides a non-negative result. Similarly, property (2) is derived from the commutative product of a function product. Property (3) indicates that maximum value of weight is obtained if and only if nodes *v_i_* and *v_j_* have the same similarity distribution.

These former properties stand for each scale *s*, although for the sake of simplicity this index was not included on previous notation.

Furthermore, each node *v_i_* ∈ 𝒱 contains also information on the location within the image in terms of positions, which will be useful in posterior scale aggregation steps.

On the other hand, the essence of this algorithm relies on aggregation, which consists of grouping and clustering those similar nodes/segments in subgraphs, according to some criteria along scales.

The proposed method bases the aggregation procedure on the weights in 𝒲, given the fact that, those pairs of nodes/subgraphs with higher weights are more similar than those with lower weights, and therefore, those former pairs deserve to be aggregated under a same segment/subgraph. Thus, a function must be defined to provide some order in set 𝒲, so that posterior subgraphs in subsequent scales contain nodes with high weights and, therefore, high similarity.

Let Ω be an ordering function, which orders edges in ℰ according to 𝒲, as follows:

(2) $$\begin{array}{l} \Omega :ℰ\mapsto 𝕉\\ \;\;\;\;\;\;\;e\mapsto \Omega (e) \end{array}$$

so that if 𝒲_*i*,*j*_ = 𝒲(*e*_*i*,*j*_) ≥ 𝒲_*i*,*k*_ = 𝒲(*e*_*i*,*k*_), then Ω(*e*_*i*,*j*_) ≥ Ω(*e*_*i*,*k*_).

In other words, let *e* = {*e*_1_, . . ., *e_m_*} be a set of edges. If Ω is applied to previous set *e*, then it is satisfied that Ω(*e*)*_i_* ≥ Ω(*e*)*_j_*, with *i* ≤ *j*, ∀ *i*, *j*, being Ω(*e*)*_i_* the *i*th element in the ordered set Ω(*e*). Concretely, Ω_𝒲_ represent the weight set 𝒲 after Ω is applied.

Once the concept of ordering function is introduced, the algorithm aggregates pair of nodes based on this former weight ordering, ensuring that the dispersion of each segment remains bounded. This aggregation criteria is represented by the Equation (3):

(3) $$\delta_{i,j}=\{\begin{array}{ll} 1, & \sigma_{i,j}\leq \sqrt{\sigma_{i}\sigma_{j}}\\ 0, & \text{otherwise} \end{array}$$

where *σ*_*i*,*j*_ represent the dispersion of aggregating nodes *v_i_* and *v_j_* . Despite of selecting the geometric mean as the comparison criteria in previous equation, other methods are possible such as arithmetic mean, generalized mean or harmonic mean. The selection of geometric mean was carried out based on experimental results.

Once pairs of nodes have been ordered and an aggregation criteria have been stated, the Gaussian Multiscale Algorithm aggregates pair of nodes with previous criteria Equation (4), considering the fact that ${𝒢}_{v_{i}}^{\left[s\right]}$ represents the *n*-th graph in scale *s*, so that $v_{i}\in {𝒢}_{n}^{\left[s\right]}$.

(4) $$\begin{array}{c} \left({𝒢}_{v_{i}}^{\left[s\right]},{𝒢}_{v_{j}}^{\left[s\right]}\right)=\\ \{\begin{array}{ll} \left({𝒢}_{p}^{\left[s\right]}\delta_{\sigma_{i,j}}+{𝒢}_{p+1}^{\left[s\right]}{\bar{\delta }}_{\sigma_{i,j}},{𝒢}_{p}^{\left[s\right]}\right) & ∄n/v_{i}\in {𝒢}_{n}^{\left[s\right]},∄m/v_{j}\in {𝒢}_{m}^{\left[s\right]}\\ \left({𝒢}_{n}^{\left[s\right]},{𝒢}_{n}^{\left[s\right]}\delta_{\sigma_{i,j}}+{𝒢}_{p}^{\left[s\right]}{\bar{\delta }}_{\sigma_{i,j}}\right) & v_{i}\in {𝒢}_{n}^{\left[s\right]},∄m/v_{j}\in {𝒢}_{m}^{\left[s\right]}\\ \left({𝒢}_{m}^{\left[s\right]}\delta_{\sigma_{i,j}}+{𝒢}_{p}^{\left[s\right]}{\bar{\delta }}_{\sigma_{i,j}},{𝒢}_{m}^{\left[s\right]}\right) & v_{j}\in {𝒢}_{m}^{\left[s\right]},∄n/v_{i}\in {𝒢}_{n}^{\left[s\right]}\\ {𝒢}_{n}^{\left[s\right]},{𝒢}_{m}^{\left[s\right]} & v_{i}\in {𝒢}_{n}^{\left[s\right]},v_{j}\in {𝒢}_{m}^{\left[s\right]} \end{array} \end{array}$$

In other words, GMA approach aggregates a pair of nodes in scale *s* under the same existing segment when at least one of both is already assigned to a segment. In case none has previously assigned to any segment, a new segment is provided. In all previous cases, aggregation is carried out as long as *δ*_*i*,*j*_ holds, otherwise, different segments are assigned to previous pair of nodes.

In addition, the number of assigned graphs in scale *s* is given by *p*, whose description is provided in Equation (5), which depends on *δ̄*_*i*,*j*_ = 1 − *δ*_*i*,*j*_ as follows:

(5) $$p=p+{\bar{\delta }}_{i,j}+\xi_{i,j}$$

where function *ξ*_*i*,*j*_ is defined as

(6) $$\xi_{i,j}=\{\begin{array}{ll} {\bar{\delta }}_{i,j}, & ∄n,m,\in \mathbb{N},m\neq n,v_{i}\in {𝒢}_{n}^{\left[s\right]},v_{j}\in {𝒢}_{m}^{\left[s\right]}\\ 0 & \text{otherwise} \end{array}$$

This assignment is done for each value in the ordered set Ω_𝒲_, until whether every element in Ω_𝒲_ is evaluated or every node in 𝒱 is assigned a segment in subsequent scale.

Gaussian Multiscale Aggregation assures that every node in scale *s* − 1 is assigned a segment/subgraph in scale *s*.

After aggregation, nodes in scale *s* are gathered into *p* subgraphs, with *p* < *N*^[^*^s^*^]^, being *N*^[^*^s^*^]^ the number of nodes in scale *s*. Each subgraph contains a set of nodes, whose number is unknown a priori. These subgraphs must be compared in subsequent scales, and thus the similarity function in subgraphs is defined in Equation (7).

Consequently, let ${𝒢}_{n}^{\left[s+1\right]}$ be the *n*th aggregated graph in scale *s* + 1, which gathers a set of *m* subgraphs { ${𝒢}_{1}^{\left[s\right]}$, . . ., ${𝒢}_{m}^{\left[s\right]}$} in scale *s*. Then the similarity function for graph ${𝒢}_{n}^{\left[s+1\right]}$, namely $\varphi_{{𝒢}_{n}^{\left[s+1\right]}}$ is defined as

(7) $$\varphi_{{𝒢}_{n}^{\left[s+1\right]}}=\frac{\sum_{i=1}^{m}\varphi_{{𝒢}_{i}^{\left[s\right]}}}{\int_{\alpha }\sum_{i=1}^{m}\varphi_{{𝒢}_{i}^{\left[s\right]}}d\alpha }$$

Notice that the definition of the similarity functions $\varphi_{{𝒢}_{n}^{\left[s+1\right]}}$ has sense also for individual nodes in 𝒱, considering nodes as graphs of one element. This is essential during the aggregation in first scale, where graphs gathers nodes instead of subgraphs. In this case, function *φ*^[0]^ is represented by a gaussian function of mean and deviation corresponding to the average and dispersion intensity of their neighbour nodes, as stated before.

Therefore, similarity functions can be completely defined as in Equation (8)

(8) $$\varphi_{{𝒢}^{\left[s+1\right]}}=\{\begin{array}{ll} 𝒩(\mu ,\sigma ) & s=0\\ \frac{\sum_{i=1}^{m}\varphi_{{𝒢}_{i}^{\left[s\right]}}}{\int_{\alpha }\sum_{i=1}^{m}\varphi_{{𝒢}_{i}^{\left[s\right]}}d\alpha } & s>0 \end{array}$$

where 𝒩(*μ*, *σ*) stands for the gaussian distribution given an average *μ* and a *σ*, both of them corresponding to their respective neighbour properties. For clarity sake, first scale (*s* = 0) is obtained based on nodes *v* ∈ 𝒱 and subsequent scales are obtained by gathering subgraphs.

Concerning location, the position of subgraphs is obtained by averaging the position of the nodes contained on each subgraph. This is essential in order to provide a neighbourhood structure, since after aggregation every scale *s* collects a scatter set of subgraphs. This structure is given by means of Delaunay triangulation.

A Delaunay graph for a set *S* = {*p*_1_, . . ., *p_n_*} of points in the plane has the set *S* as its vertices. Two vertices *p_i_* and *p_j_* are joined by a straight-line (representing an edge) if and only if the Voronoi regions *V* (*p_i_*) and *V* (*p_j_*) share an edge. In addition, for a set of points in 𝕉^2^, knowing the locations of the endpoints permits a solution in *O*(*nlogn*) time. Therefore, Delaunay triangulation is a suitable method to provide a neighbourhood structure to previous aggregated subgraphs.

This operation represents the final step in the loop, since at this moment, there exist a new subgraph ${𝒢}^{\left[s+1\right]}=\cup_{k}{𝒢}_{k}^{\left[s+1\right]}$ at scale *s* + 1 where each ${𝒢}_{k}^{\left[s+1\right]}$ represents a node, and edges ℰ^[^*^s^*^+1]^ are provided by Delaunay triangulation, and weights 𝒲^[^*^s^*^+1]^ are obtained based on Equations (1) and (8).

The whole loop is repeated until only two subgraphs remain, as stated at the beginning of this section. However, due to the constraints provided to aggregate (Equation (9)), the method could not aggregate more segments, without achieving the goal of dividing image into two subgraphs. Therefore, Equation (3) is in practice relaxed and stated as follows in Equation (9):

(9) $$\sigma_{i,j}^{\left[s+1\right]}\leq \sqrt{\sigma_{i}^{\left[s\right]}\sigma_{j}^{\left[s\right]}}+k^{\left[s\right]}$$

being *k*^[^*^s^*^]^ a factor able to avoid aggregation method from being stuck in the loop. This factor can be dynamically increased or decreased, according to previous method necessities. However, this value is initially set to *k*^[^*^s^*^]^ = 0.01, for each scale *s*. The capability of *k*^[^*^s^*^]^ to adapt the necessities of the algorithm remains as future work.

The computational cost of this algorithm is quasi-linear with the number of pixels, since each scale gathers nodes in the sense that nodes in subsequent scales are reduced by (in practice) a three times factor (Figure 4). Therefore, time to process the first scale (which contains the highest number of nodes) is greater than the rest of times to process subsequent scales, and the total time is approximately comparable to two times the processing time to aggregate first scale. This statement will be supported within the results of Section 5.

## 4. Database

After presenting the algorithm, next section describes the creation of the database involved in evaluation.

This section describes the creation of a synthetic database containing a total of 408,000 images of hands with a wide range of possible backgrounds like carpets, fabric, glass, grass, mud, different objects, paper, parquet, pavement, plastic, skin and fur, sky, soil, stones, tiles, tree, wall and wood.

The main aim of this database is twofold:

- First, the main purpose is to provide a comparative evaluation frame for segmentation algorithm, where existing approaches in literature could be compared. In other words, this database makes it possible to assess to what extent the segmentation algorithm can satisfactory perform a hand isolation from background on real scenarios.
- In addition, this database contains the ground-truth result for each image, providing a possible supervised evaluation criteria. These ground-truth images were obtained, given that hands were taken with a blue-coloured background, so that hand can be easily extracted by simple thresholding [22].

The creation of the synthetic database (named GB2S Database) considers the hands extracted in former database and the set of the aforementioned different textures, which were obtained from the website *http://mayang.com/textures/*.

First of all, a straightforward segmentation was carried out with a threshold-based segmentation [22], obtaining two binary masks: *M_h_*, corresponding to those pixels representing hand, and *M_b_* with pixels corresponding to background.

Afterwards, both masks are laid one over each other, with *M_b_* containing pixels associated to a specific texture, resulting in an image with the hand over a desired background (grass, water, wood and so forth).

In order to ensure there is no considerable difference in illumination between hand and background, each image is converted from RGB to YCbCr color space [9] carrying out a histogram equalization in terms of illumination (Y), performing afterwards the inverse transform from YCbCr to RGB color space. Finally, a morphological operation consisting on an opening operator with a structural element of a disk of small size (5 pixels radio) is considered to fade the boundary between hand and background, so that hand is integrated within background.

All these former operations attempt to ensure a fair scenario, simulating the conditions provided in real situations.

**Figure 2.**
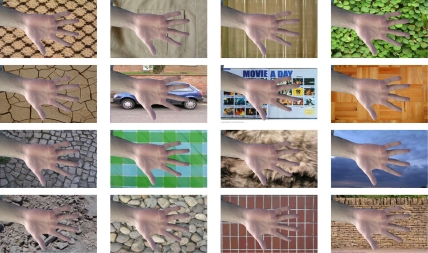
Samples from the synthetic database in different backgrounds for a given acquisition.

For each hand image, a total of 5 × 17 (five images and 17 textures) synthetic images are created, collecting a total of 120 × 2 × 20 × 5 × 17 = 408,000 images (120 individuals, two hands, 20 acquisitions per hand, five images and 17 textures) to properly evaluate segmentation on real scenarios. Some visual examples of this database are provided in Figure 2.

This presented database is publicly available at http://www.gb2s.es/.

Once the database has been presented, the following section comes out with the evaluation of the algorithm and the obtained results.

## 5. Results and Discussion

This section contains the results of the comparative evaluation of the proposed approach to LDC [7] and NCut [8]. First, the evaluation criteria is stated in order to provide a comparative frame, providing afterwards the results obtained in the evaluation.

### 5.1. Evaluation Criteria

Although there exist some unsupervised evaluation methods for image segmentation [32,34,35], we have preferred a supervised segmentation, since the synthetic database GB2S contains the corresponding ground-truth associated to each image. Segmentation results will be compared to this ground-truth image.

The proposed evaluation method is based on F-measure, [36], defined as follows:

(10) $$F=\frac{2\mathrm{RP}}{R+P}$$

where *P* (Precision or Confidence) stands for the number of true positives (true segmentation, *i.e.*, classify a hand pixel as hand) in relation to the number of true positives and false negatives (false hand segmentation), and *R* (Recall or Sensitivity) represents the number of true positives in relation to the number of true positives and false positives (false background segmentation, *i.e.*, consider background as hand). F-measure is within the [0, 1] interval, so that 0 states a bad segmentation, while on the contrary 1 represents the best segmentation result.

Aiming a fair comparison, the propose algorithm is compared to two competitive segmentation methods existing in the literature, namely Lossy Data Compression (LDC) [7] and Normalized Cuts (NCut) [8].

### 5.2. Gaussian Multiscale Aggregation Evaluation

The evaluation of a segmentation method involves different aspects concerning accuracy, computational cost and parameters dependency.

First aspect is related to what extent the algorithm is able to properly detect or isolate a specific object within an image. Concretely in this paper, accuracy is understood as the capability of the proposed algorithm to properly isolate hand from background.

Table 1 shows the results in terms of F-measure of the proposed methods in comparison to LDC approach and Normalized Cuts. Although the results obtained by the proposed method (first column) can be improved, they overcome the other two schemes. Reader may notice that scenarios with textures similar to hand (e.g., soil) decrease the performance of the segmentation algorithms, but the proposed method still provides F-measure rates of more than 88%.

**Figure 3.**
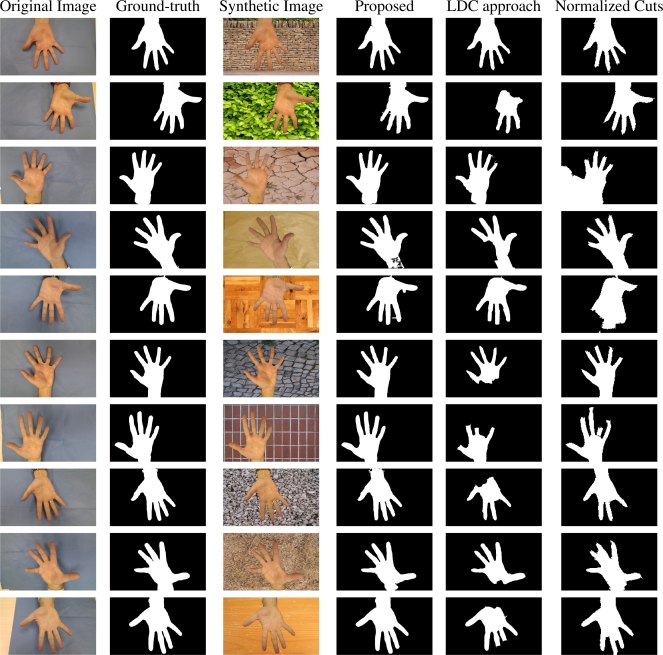
A comparative study of results provided by segmentation algorithm in comparison to ground-truth. First column gathers examples from first database, together with their segmentation on second column, considered as ground truth. Third column presents synthetic images based on first column images, providing on the fourth column the final segmentation result. Last two column present the segmentation result provided by the Lossy Data Compression (LDC) [7] and Normalized Cuts [8], respectively.

In addition, accuracy can be also visually evaluated. Figure 3 presents a comparative frame for segmentation evaluation, comparing the results obtained for the LDC method, Normalized Cuts and the proposed method. Reader can compare the obtained results (columns 4–6) to the ground-truth (column 2). The results obtained by the proposed approach conserve more precisely the shape of the hand even in scenarios with similar textures like parquet (row 5) or wood (last row).

Secondly, concerning computational cost, Table 2 presents the segmentation time in relation to the number of pixels of the images. This temporal evaluation was carried out in a PC computer @2.4 GHz Intel Core 2 Duo with 4 GB 1,067 MHz DDR3 of memory, considering that the proposed method was completely implemented in MATLAB.

The results provided in Table 2 shows that the proposed algorithm is faster than the compared approaches. In addition, the proposed method can segment images of higher sizes, but LDC and NCut cannot handle higher sizes images without running out of memory.

Finally, this section will study the dependency of two parameters strongly related to algorithm performance, namely *k* factor and aggregation linearity (Equations (9) and (10)).

Factor *k* controls the aggregation capability of the overall method. Within these experiments, factor *k* was experimentally fixed to *k* = 0.01, ensuring that the number of segments in the last scale is two: hand and background. However, extending the proposed approach to other applications in image processing would imply to provide a dynamic factor *k*, depending whether the algorithm standstills in a certain scale. The proposal of a dynamic factor *k* remains as future work.

**Figure 4.**
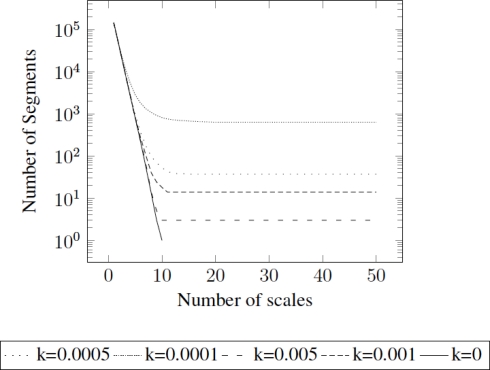
Dependency of the aggregation process on parameter *k*. The lower *k*, the lower the constraints to aggregate segments. Notice that *k* = 0 means no stopping condition.

Figure 4 presents the relation between number of segments along scales using different values of *k*. Notice that *k* = 0 implies no stopping criteria, and therefore aggregates scales until only one segment is obtained.

**Figure 5.**
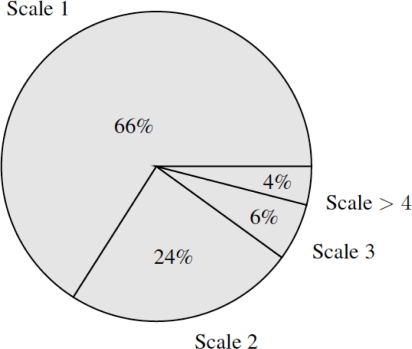
Proportion of processing time for each scale. Most of the time is required by the aggregation procedure on the first scale.

During the explanation of the method, the algorithm is said to be quasi-linear with the number of pixels. This statement is supported by Table 2, but for clarity sake, we provide a chart (Figure 5) indicating which proportion of time is required for each scale. The most demanding scale is the first one, whose proportion is higher than the other parts, concluding that the algorithm has indeed a quasi-linear behaviour in relation to the number of pixels.

## 6. Conclusions and Future Work

The application of hand biometrics to unconstrained and contact-less, platform-free environments implies an increase in difficulty in the pre-processing and segmentation procedure in hand acquisition. Therefore, an unsupervised segmentation algorithm has been proposed based on Gaussian multiscale aggregation. This method gathers iteratively those pixels similar in texture and color under segments, until a certain number of clusters/segments is provided as a result.

This method is able to isolate hand from a wide range of backgrounds (carpets, fabric, glass, grass, mud, different objects, paper, parquet, pavement, plastic, skin and fur, sky, soil, stones, tiles, tree, wall and wood), simulating real situations and unconstrained background scenarios.

Besides, the evaluation of the proposed approach has been carried out based on a publicly available synthetic database, containing 408,000 hand image acquisitions with different background textures. The evaluation consisted of a comparison of the performance in terms of accuracy and computational cost to two competitive segmentation methods existing in literature, namely Lossy Data Compression (LDC) [7] and Normalized Cuts (NCuts) [8].

The results obtained point out that the performance of the proposed algorithm outcomes existing segmentation algorithms in literature, regarding not only accuracy and computational cost, but also memory usage, since the proposed algorithm is quasi-linear in relation to the number of pixels.

As future work, we consider to implement the method with a dynamic *k* parameter, so that the algorithm can be adapted to any image, providing segmentations of more complex images. In addition, we aim a faster implementation of the method considering both software and hardware optimized implementation, together with a more complete evaluation with other publicly available databases.

## Figures and Tables

**Table 1.** Segmentation evaluation by means of F-measure in database GB2S with 17 different background textures, together with the corresponding standard deviation. In addition, the results for LDC and NCut are also provided for comparison.

Texture	Proposed, *F* (%)	LDC, *F* (%)	NC, *F* (%)
Carpets	92.1 ± 0.1	73.7 ± 0.3	65.1 ± 0.3
Paper	91.3 ± 0.1	83.2 ± 0.2	72.8 ± 0.4
Stones	91.2 ± 0.1	78.2 ± 0.4	71.5 ± 0.3
Fabric	88.4 ± 0.3	65.3 ± 0.1	60.1 ± 0.2
Parquet	88.3 ± 0.2	66.1 ± 0.2	62.3 ± 0.3
Tiles	90.1 ± 0.2	71.5 ± 0.3	68.7 ± 0.2
Glass	94.1 ± 0.1	75.8 ± 0.1	71.4 ± 0.1
Pavement	88.9 ± 0.2	67.8 ± 0.1	63.7 ± 0.2
Tree	96.0 ± 0.2	73.4 ± 0.2	67.2 ± 0.1
Grass	93.3 ± 0.2	70.1 ± 0.1	65.3 ± 0.2
Skin and Fur	95.3 ± 0.3	82.3 ± 0.2	71.8 ± 0.3
Wall	94.1 ± 0.1	70.9 ± 0.2	62.3 ± 0.2
Mud	89.5 ± 0.2	68.3 ± 0.1	60.1 ± 0.2
Sky	96.1 ± 0.1	77.2 ± 0.2	71.3 ± 0.1
Wood	93.5 ± 0.1	82.5 ± 0.2	73.5 ± 0.1
Objects	92.0 ± 0.1	70.1 ± 0.1	61.6 ± 0.3
Soil	89.0 ± 0.2	67.2 ± 0.3	59.7 ± 0.2

**Table 2.** Relation between time performance (in seconds), the dimension of the image, and the size in number of pixels, comparing the proposed method with LDC approach and Normalized Cuts (NCut).

Image Dimensions	Number of Pixels	Proposed (seconds)	LDC (seconds)	NCut (seconds)

600 × 800	480,000	30.1	233.1	321.7
450 × 600	270,000	19.8	63.4	129.5
300 × 400	120,000	9.4	52.1	25.1
150 × 200	30,000	3.1	32.8	7.2
